# Soil Salinity Drives the Arbuscular Mycorrhizal Fungal Generalists and Specialists Subcommunity Assembly in Extremely Dryland Forest in China

**DOI:** 10.3390/microorganisms13081742

**Published:** 2025-07-25

**Authors:** Mengjun Qu, Jianming Wang, Yin Wang, Xuge Zou, Xun Lei, Meiwen Luo, Wenkai Wang, Jingwen Li

**Affiliations:** 1 School of Ecology and Nature Conservation, Beijing Forestry University, Beijing 100083, China; qumengjun@bjfu.edu.cn (M.Q.); lei3220729@bjfu.edu.cn (X.L.); lmw123@bjfu.edu.cn (M.L.); 18435387562@163.com (W.W.); 2 State Key Laboratory of Efficient Production of Forest Resources, Beijing Forestry University, Beijing 100083, China; 3 Hebei Collaborative Innovation Center for Eco-Environment, College of Life Sciences, Hebei Normal University, Shijiazhuang 050024, China; wangyin@hebtu.edu.cn; 4 Henan Danjiang Wetland National Nature Reserve Management Office, Nanyang 474450, China; zzxg123@bjfu.edu.cn

**Keywords:** soil salinity, AM fungi, community assembly, dryland forest, ecological network

## Abstract

AM fungi play a pivotal role in regulating ecosystem functioning and processes. However, the assembly of soil AM fungal communities and its drivers across *Populus euphratica* forests in extremely arid regions remain largely unclear. Here, we explored the composition and assembly processes of AM fungal communities in the soil of *P. euphratica* forests in northwest China. The results showed that soil salinity affected the composition, assembly processes, and network stability and complexity of AM fungal communities. Stochastic processes rather than deterministic processes dominated the community assembly of AM fungi. Habitat generalists were more susceptible to deterministic processes than specialists. In addition, the network analysis showed that fungal network complexity had a hump-shaped relationship with increasing soil salinity, while network stability had a U-shaped relationship. This research suggests that soil salinity plays an essential role in determining AM fungal community composition and assembly processes in *P. euphratica* forests of arid regions.

## 1. Introduction

Microorganisms are among the most diverse biological resources on the Earth, including fungi, bacteria and archaea. Multiple studies have shown that soil microorganisms, as important components of ecosystems, exert a significant influence on ecosystem processes and functions such as material and nutrient cycling [1,2]. Soil microbial communities are generally composed of several specialists and generalists, both of which have important effects on microbial community dynamics [3]. Specifically, specialists have narrow habitat niches and specific environmental adaptability, while generalists have a wide range of environmental tolerance and display considerable flexibility in metabolism [4]. As a result, both of them exhibit different assembly processes due to their differences in environmental adaptability. AM fungi are the most important endomycorrhizal fungi among soil microorganisms, which live in the soil and plant roots [5], not only providing access to plant photosynthesis products but also supplying mineral nutrients to plants through an external mycelial network [6,7]. About 80% of terrestrial plants have a capacity to form symbiotic relationships with these fungi [7]. Thus, AM fungi are essential for the equilibrium and functionality of ecosystems [8], as they take part in and modify a wide range of ecological processes, including acquisition of resources, regulation of plant community composition, intraspecific plant competition [9], response to both biotic and abiotic stresses [10,11,12], ecosystem succession [13], soil structure formation [14], and soil nutrient cycling [7]. However, current research on soil microorganisms focuses on two major microbial groups, bacteria and fungi, and the composition and assembly of AM fungal communities need further exploration.

The critical role of soil microorganisms in biogeochemical cycling has prompted extensive research on microbial community composition and assembly in recent decades [15,16]. Moreover, revealing the ecological processes that drive community construction has been one of the main concerns in ecological research [17]. The niche theory emphasizes the importance of deterministic processes, such as biological and environmental filtration, while the neutral theory states that stochastic processes or dispersal limitation is crucial to community assembly [18,19]. An issue that has attracted attention is the relative contribution of deterministic and stochastic processes to AM fungal community assembly [8,20]. Actually, both deterministic and stochastic processes shape the assembly of AM fungal communities [5]. It has been shown that deterministic factors, including soil chemistry and nutrient availability, light intensity, precipitation, pH, host plants, and anthropogenic disturbance, play a key role in AM fungal community assembly [5]. Numerous studies have indicated that ecological processes that determine the composition of AM fungal communities differ depending on scales and host plants [5,8,21]. Nevertheless, the assembly mechanisms of microbial generalist and specialist subcommunities remain controversial. A study on bacteria and fungi in the rhizosphere of *Populus euphratica* has revealed that the community assembly of specialists and generalists was dominated by stochastic processes [4]. A study on farmland soil microbiome has suggested that the community assembly of specialists is significantly controlled by deterministic processes, while that of generalists is dominated by stochastic processes [22]. Moreover, another study has reported that stochastic processes have significant impacts on both bacterial generalists and specialists, but it is worth noting that specialists play a more critical role in maintaining the stability of microbial network [23]. Currently there is a lack of agreement on AM fungal community assembly mechanisms and network characterization in the arid regions of northwest China, and this area remains poorly explored.

*Populus euphratica* Oliv. (Salicaceae) is an important component of arid and semi-arid ecosystems, and it is listed as a rare and endangered species worldwide [24]. However, soil salinization in the oasis shows a trend of intensification due to climate change, human activity, and a severe lack of water resources in the arid region of northwest China [25,26], and oasis plants have long been subjected to drought and salinity–alkali stress. Currently, *P. euphratica* in northwest China is threatened by the risks of population decline and regeneration failure. *P. euphratica* is a key mycorrhizal plant whose roots are able to develop a symbiotic relationship with AM fungi in the soil [27,28]. Studies have shown that AM fungi have the capacity to improve rhizosphere soil conditions, regulate plant uptake of water and nutrients, resist planet physiological drought, promote plant growth and production, and, thus, improve the saline–alkali tolerance of plants. Specifically, this is mainly achieved by forming a mycelial network which enables plants to extend their utilization range to surrounding soils and expand the absorption area and spatial extent of the roots [29,30]. Moreover, studies have shown that plants can attract specific beneficial soil microorganisms in response to high-salinity soil conditions [31,32]. Nonetheless, it has not been systematically studied how AM fungal communities (including specialists and generalists) respond to soil salinity changes in *P. euphratica* forests in arid areas.

To fill the gap in knowledge, we investigated the composition and assembly of the AM fungal community and their drivers in *P. euphratica* forests in northwest China. We proposed the following hypotheses: (1) stochastic processes dominate the community assembly of AM fungal communities (including specialists and generalists) in *P. euphratica* forests; (2) compared with generalists, specialists are more susceptible to deterministic processes; and (3) soil salinity will reduce the stability of AM fungal networks.

## 2. Materials and Methods

### 2.1. Study Region and Sample Collection

The present study was conducted in the arid zone of northwest China, where *P. euphratica* is mainly distributed. Because of the great distance from the ocean and the obstruction of the easternmost Helan Mountain, as well as the Tibetan Plateau, Kunlun Mountains, Altun Mountains, and Qilian Mountains in the south, the northwest arid zone has a temperate continental climate with little rainfall [33]. At the start of the 21st century, the average annual temperature is 8.63 °C, with an annual precipitation of 156.36 mm [34], making it one of the world’s driest regions. As a result, the composition of *P. euphratica* forests is relatively simple, with shrub plants, such as *Tamarix chinensis*, *Lycium ruthenicum*, and *Alhagi camelorum*, and herbaceous plants, such as *Sophora alopecuroides*, *Aeluropus pungens*, *Glycyrrhiza inflata*, and *Phragmites australis*.

**Figure 1:**
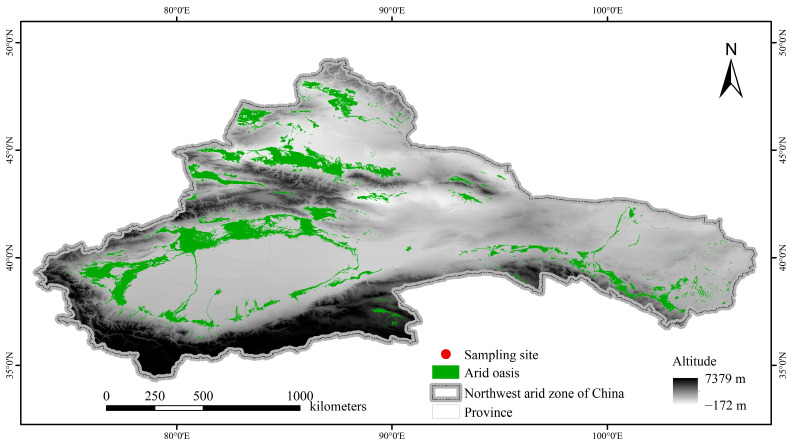
Distribution of 11 sampling sites in the northwest arid zone of China.

We selected a total of 11 sites (Figure 1), including 9 in Xinjiang Autonomous Region, 1 in Inner Mongolia Autonomous Region, and 1 in Gansu Province, with latitude and longitude ranging from 38.07° N to 44.61° N and 79.53° E to 101.09° E, as well as the longest span of 1816.3 km from east to west. Within each site, 4–6 quadrats (20 m × 30 m) were randomly established under representative landscapes and dominant vegetation, and finally a total of 54 quadrats were established. GPS coordinates, altitude, and plant community richness (PR) were recorded at each quadrat. Approximately 20–30 soil cores were collected at a depth of 0–10 cm under plant canopy within each quadrat with a soil drill and mixed thoroughly to obtain a final total of 54 soil samples. After removing roots, stones, and litter with a 2 mm mesh, the soil samples were divided into the following two groups: one group was kept at 4 °C to determine the physical and chemical properties, while the other was kept at −20 °C to sequence DNA. In addition, soil samples were taken using the ring drill method (100 cm^3^) to measure soil moisture and bulk density in each quadrat.

### 2.2. Soil, Climate, and Vegetation Factors

In the lab, bulk density (BD, g/cm^3^) and soil moisture content (SMC, %) were measured by drying the soil samples at 105 °C until a steady weight was obtained. Soil samples were used to evaluate physical and chemical properties after being exposed to airing and drying. Subsequently, soil pH was calculated at a soil-to-water ratio of 1:2.5, and soil conductivity was determined in a 1:5 soil:water mixture (salinity, mS/cm). Soil organic carbon content (SOC, g/kg) was determined by the K_2_Cr_2_O_7_ oxidation method. Total nitrogen content (TN, g/kg) was measured by the Kjeldahl method, and total phosphorus content (TP, g/kg) was measured by the molybdenum blue method.

Mean annual temperature (MAT), precipitation seasonality (PS), and mean annual precipitation (MAP) data were obtained from the WorldClim database (https://www.worldclim.org, accessed on 19 March 2025). Aridity index (AI) and potential evapotranspiration (PET) data were obtained from the Global-AI_PET_v3 database [35]. Normalized difference vegetation index (NDVI) data were obtained from the National Ecosystem Science Data Center (http://www.nesdc.org.cn, accessed on 19 March 2025) [36].

### 2.3. DNA Extraction and Sequencing

After melting the soil samples on ice, centrifuging, and mixing thoroughly, the samples were checked using a Thermo Scientific NanoDrop Spectrophotometer (Waltham, MA, USA), and 30 ng was taken for PCR amplification. The primers LR1 (5’-GCATATCAATAAGCGGAGGA-3’) and FLR2 (5’-GTCGTTTAAAGCCATTACGTC-3’) [37] were subjected to a first round of PCR, and the final 25 μL reaction mixtures contained 1 μL of each primer (5 μM), 3 μL of bovine serum albumin (BSA, 2 ng/μL), 12.5 μL of 2xTaq Plus Master Mix, and 7.5 μL of ddH2O. Initially, the PCR protocol was as follows: 5 min of 94 °C denaturation, 30 cycles of 94 °C denaturation for 30 s, 30 s of annealing at 58 °C, 60 s of 72 °C extension, and a final extension at 72 °C for 7 min. The second round of PCR used the primers FLR3 (5’-TTGAAAGGGAAACGATTGAAGT-3’) and FLR4 (5’-TACGTCAACATCCTTAACGAA-3’) [38]. The template was changed using the first amplification’s product, and the reaction mixtures and cycling parameters were the same as those used in the first round. PCR products were purified using the Agencourt AMPure XP Nucleic Acid Purification Kit (Axygen Biosciences, Union City, CA, USA) and analyzed by 1% agarose gel electrophoresis to determine the size of the amplification target bands. After purification, the amplicons were combined in equimolar concentrations and sequenced on an Illumina MiSeq platform (San Diego, CA, USA).

### 2.4. Analysis of Habitat Specialists and Generalists

The generalists and specialists were classified according to a recent study [4]. If the species enrichment occurred in narrow environments, these species were defined as specialists (e.g., enriched in limited environments), while generalists were species enriched in wide environments [22]. We used Levin’s niche breadth width to determine the habitat specialization of soil AM fungi in *P. euphratica* forests with the “niche.width” function in the “spaa” package (version 0.2.2, https://CRAN.R-project.org/package=spaa, accessed on 19 March 2025) [39], which reflects the range of environmental adaptability of the growth and reproduction of species. Specially, a species with a wider niche breadth generally has greater environmental adaptability and is more likely to survive on larger temporal or spatial scales. In contrast, lower niche breadth values indicate that the OTUs occurred in fewer habitats and were unevenly distributed. Generalist species have wider fundamental niches than do specialists [22]. Afterwards, we identified microbial generalists and specialists and calculated the occurrences of species generated with the “EcolUtils” package (version 0.1, https://github.com/GuillemSalazar/EcolUtils, accessed on 19 March 2025) [40]. The AM fungi were labeled as habitat generalists or specialists according to the occurrence of each particular OTU in this particular environment. When habitat-specialization species values for this study area exceeded the upper 95% confidence interval, AM fungi species were identified as habitat generalists; the AM fungi species whose observed occurrence fell below the lower 95% confidence interval of the 1000 permutations were identified in this study area as habitat specialists [4].

### 2.5. Null Model and Neutral Model Analysis

Before performing community assembly processes, phylogenetic Mantel correlogram was used to examine phylogenetic signals (Figure S1). AM fungal community assembly processes were assessed using the null model-based β diversity metrics Raup–Crick (RC_Bray_) and β-nearest taxon index (βNTI) [4]. Specifically, stochastic and deterministic processes are dominant when |βNTI| < 2 and > 2. In brief, βNTI < −2 indicates that the coexisting community (target community) is significantly lower than the phylogenetic turnover, that is, the homogeneous selection processes, and βNTI > 2 indicates that the coexisting community is significantly higher than the phylogenetic turnover, that is, the variable selection processes. When −2 < βNTI < 2, −0.95 < RC_Bray_ < 0.95 represents undominated processes, RC_Bray_ > 0.95 represents dispersal limitation, and RC_Bray_ < −0.95 represents homogenizing dispersal. In addition, the relationship between taxonomic frequencies and abundances was predicted by fitting a neutral community model [41].

### 2.6. AM Fungal Network Construction and Stability Analysis

To map the AM fungal network, we screened OTUs with a relative abundance greater than 0.1%, and constructed the network for five salinity levels using Spearman correlation coefficients. The “rcorr” function in the “Hmisc” package (version 5.2-3, https://CRAN.R-project.org/package=Hmisc, accessed on 19 March 2025) [42] was used for pairwise comparisons based on OTUs, and *p*-values were adjusted accordingly. To better quantify the topological properties of the AM fungal network for each salinity level, the “igraph” package (version 2.1.4, https://CRAN.R-project.org/package=igraph, accessed on 19 March 2025) [43] was used to construct the ecological network, where each node represented an OTU and each edge represented a strong and significant correlation. The resulting network was visualized using the interactive platform Gephi (0.10.1). Furthermore, robustness and vulnerability metrics were used to characterize the stability of the AM fungal network [44,45,46]. Both were based on a simulated network, where robustness was defined as the proportion of species remaining in the network after random or targeted removal of nodes; vulnerability of each node measured the relative contribution of that node to global efficiency; and vulnerability of the network is expressed in terms of the maximal vulnerability value of the nodes in the network (Table S1). In addition, the cohesion index was used to characterize the complexity of the AM fungal network [47].

### 2.7. Statistical Analysis

A total of 15 environmental variables (altitude, SMC, BD, pH, Salinity, SOC, TN, TP, MAT, MAP, PS, PET, AI, NDVI, and PR) were involved in this study. The relationships among the above variables were assessed using spearman correlation analysis. Meanwhile, the relationships between the AM fungal community composition and environmental variables were assessed using the Mantel test. Nonmetric multidimensional scaling (NMDS) analysis was used to visualize the differences in the composition of AM fungal communities among sites using the “metaMDS” function. Additionally, the “adonis” function was applied to perform a permutational multivariate analysis of variance (PERMANOVA) in order to identify any significant differences in the AM fungal community. Additionally, to explore the mechanisms by which environmental variables influence soil extracellular enzyme activity and microbial resource limitation, all variables were categorized and analyzed based on a partial least squares path modeling (PLS-PM) with the “plspm” package (version 0.4.9, https://github.com/gastonstat/plspm, accessed on 19 March 2025) [48].

## 3. Results

### 3.1. Composition of AM Fungal Community at Different Salinity Levels

**Figure 2:**
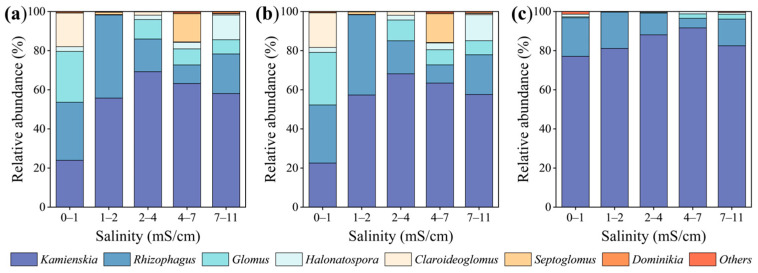
Relative abundance of the AM fungal genus at different salinity levels: (**a**) total AM fungi; (**b**) specialist AM fungi; (**c**) generalist AM fungi.

**Figure 3:**
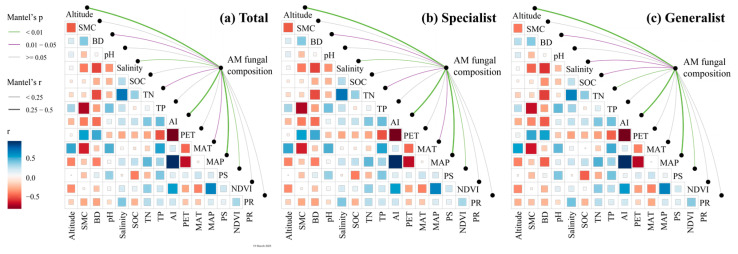
Relationships between soil AM fungal composition and environmental variables: (**a**) total AM fungi; (**b**) specialist AM fungi; (**c**) generalist AM fungi. SMC, soil moisture content; BD, soil bulk density; SOC, soil organic carbon content; TN, soil total nitrogen content; TP, soil total phosphorus content; AI, aridity index; PET, potential evapotranspiration; MAT, mean annual temperature; MAP, mean annual precipitation; PS, precipitation seasonality; NDVI, normalized difference vegetation index; PR, plant community richness.

Based on the known fungal sequences in AM fungal databases, 428 OTUs were grouped into three orders, four families, and 12 genera (Figure 2 and Figure S2). A total of 38 OTUs were classified as generalists, accounting for 8.87% of the relative abundance, while 87 OTUs were classified as specialists, accounting for 20.32% of the relative abundance. Among all identified sequences, specialists and generalists, *Kamienskia* accounted for the highest genus abundance (55.4%, 54.8%, and 85.0%), followed by *Rhizophagus* (22.2%, 21.9%, and 13.0%) and *Glomus* (10.6%, 10.9%, and 1.1%). Moreover, the total AM fungal sequences and specialists exhibited similar distributions and relative abundances at the genus level (Figure S2). Furthermore, the relative abundance of *Kamienskia* exhibited a hump-shaped trend (first increasing and then decreasing) with increasing soil salinity in the generalist and specialist subcommunities (Figure 2). In addition, as shown by NMDS analysis, the total AM fungal community and specialist subcommunity showed significant structural differences across soil salinity levels, which is supported by the results of the PERMANOVA (*p* < 0.05). However, the generalist subcommunity did not display such variations (Figure S3). The Mantel test indicated a notable influence of soil salinity on the structure of arbuscular mycorrhizal fungal communities (Figure 3).

### 3.2. Ecological Assembly Processes of AM Fungal Community at Different Salinity Levels

**Figure 4:**
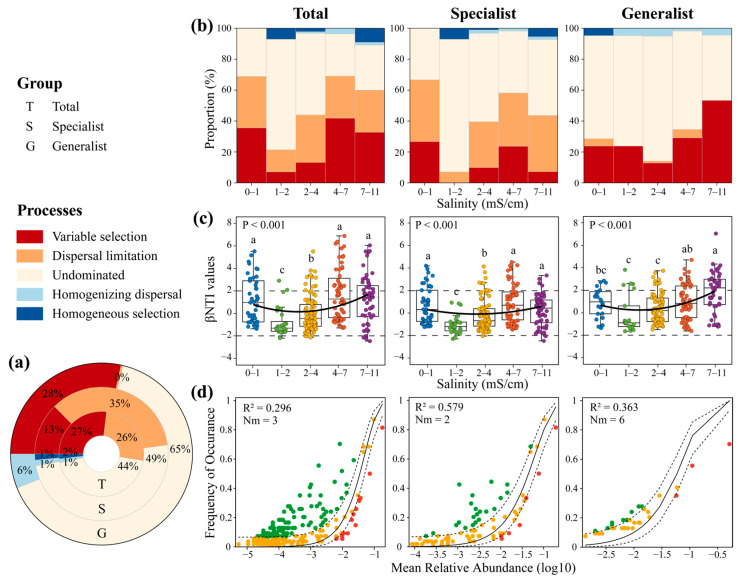
The assembly processes of the AM fungal community and the change at different salinity levels: (**a**) contribution of different ecological processes to the assembly of the AM fungal community; (**b**) different processes at different salinity levels; (**c**) variation in the βNTI at different salinity levels, where horizontal dashed lines indicate βNTI significance thresholds of +2 and −2; Boxplots that do not share a letter are significantly different (*p* < 0.05) (**d**) community assembly processes are fitted by neutral models.

Stochastic processes played a predominant role in AM fungal community assembly, accounting for 71.21%, 85.95%, and 71.54% of the contribution to the community assembly of the total AM fungi, specialists, and generalists, respectively, as evidenced by the null model analysis (Figure 4a). Moreover, all of them were primarily controlled by undominant processes. In addition, generalists were more likely to be controlled by deterministic processes than specialists. The regression analysis showed a significant correlation between soil salinity and βNTI values of the total AM fungi, specialists, and generalists (Figure S5). The impact of the dispersal limitation on specialists was found to be less significant at a salinity level of 1–2 mS/cm compared with other salinity levels. The overall effect on generalists, however, was found to be minimal across all salinity levels. Generalists were not affected by homogeneous selection at different salinity levels (Figure 4b). As shown in Figure 4c, deterministic processes (mainly variables selection) were observed in the community assembly of AM fungi, as well as specialists and generalists, at low (0–1 mS/cm) and high (7–11 mS/cm) soil salinity levels. The neutral community model was employed to elucidate the correlation between the relative abundance and frequency of occurrence of OTUs, suggesting the importance of stochastic processes in shaping AM fungal community assembly (Figure 4d).

### 3.3. Direct and Indirect Effects of AM Fungal Community Processes

**Figure 5:**
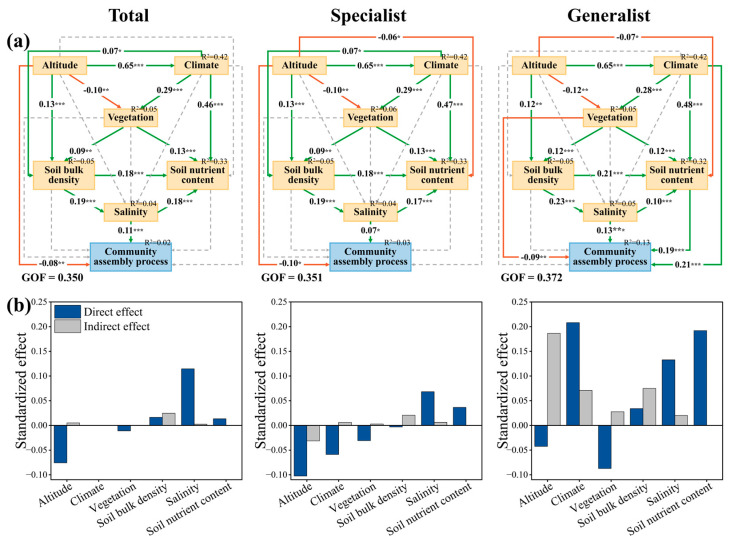
Using partial least squares path modeling (PLS-PM) to analyze the (**a**) possible pathways of the AM fungal community assembly process and (**b**) standardized effect derived from PLS-PM. Green and yellow arrows indicate positive and negative flows of causality, respectively. Numbers on the arrows indicate significant standardized path coefficients. R^2^ indicates the variance of the dependent variable explained by the models. * Represents significant effects at *p* < 0.05; ** represents significant effects at *p* < 0.01; *** represents significant effects at *p* < 0.001.

The results of the partial least squares path modeling (PLS-PM) further revealed that the soil salinity was able to affect AM fungal community assembly processes significantly and directly, which was mainly mediated by soil salinity, which itself is regulated by altitude, climate, vegetation, and soil bulk density, with the effect of soil bulk density being relatively strong. A direct negative correlation was observed between altitude and the community assembly processes of the total AM fungi and specialists, with standardized path coefficients of −0.08 and −0.10, respectively (*p* < 0.05). However, there were differences in the direct and indirect effects in the community assembly processes of generalists. Specifically, climate factors (e.g., AI, MAP, and PS) and soil nutrient contents (e.g., SOC and TN) exhibited significant positive correlations with community assembly processes, with path coefficients of 0.21 and 0.19, respectively. Additionally, soil salinity was found to have an indirect effect on community assembly processes by regulating soil nutrient contents (Figure 5). Nevertheless, the models had a large proportion of unexplained variance (Tables S2–S4), indicating that other unmeasured factors may also influence AM fungal community assembly processes.

### 3.4. Network Patterns and Stability of AM Fungal Community at Different Salinity Levels

**Figure 6:**
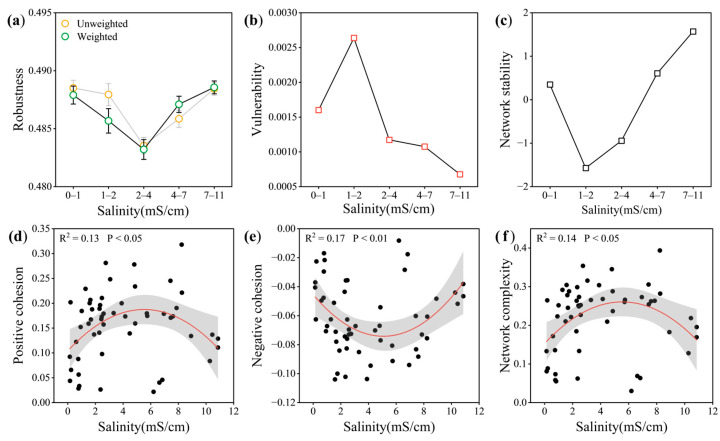
Changes in AM fungal network stability and complexity at different salinity levels. (**a**–**c**) Comparison of network stability. Robustness is measured as the proportion of taxa remaining with 50% of the taxa randomly removed from each of network. The error bar corresponds to the standard deviation of 100 repetitions of the simulation. Vulnerability is measured by the maximal node vulnerability in the network. (**d**–**f**) Relationship between network complexity and soil salinity. Network complexity is calculated as |negative| + positive cohesion. Shaded bands are the 95% CIs.

In order to determine how soil salinity affects the stability of AM fungal co-occurrence networks, the network robustness and vulnerability were evaluated. The findings showed that the network robustness exhibited a U-shaped trend as the soil salinity rose, with higher robustness observed at low and high soil salinity levels. On the contrary, the network vulnerability followed a hump-shaped trend, with lower vulnerability observed at low and high soil salinity levels (Figure 6a,b). In addition, network cohesion was used to dissect positive and negative correlations. Among them, the negative cohesion showed a quadratic linear relationship with the increase in soil salinity, and the negative cohesion of medium salinity was the lowest, but the positive cohesion showed the opposite trend (Figure 6d,e). These results implied that the AM fungal network exhibited enhanced stability at low and high soil salinity levels.

## 4. Discussion

### 4.1. AM Fungal Community Structure at Different Soil Salinity Levels

*Kamienskia* was the most dominant genus in the AM fungal community in the soil of *P. euphratica* forests, followed by *Rhizophagus* and *Glomus*. The three mentioned genera accounted for more than 85% of the total abundance. This finding challenges the results of previous studies on AM fungal communities in agroecosystems [49] and semi-arid mountain ecosystems [50], which have shown that the dominant genus is Glomus. It can be inferred that Glomus has good adaptability in different ecosystems [51]. However, as obligate symbionts, AM fungi have a community structure that is closely related to plant community characteristics and influenced by specific habitat conditions [8], so their relative abundances vary across different genera. This may indicate that *Kamienskia* was the most adaptable genus of AM fungi in *P. euphratica* forests in northwest China. In addition, AM fungi are found to have the capacity to enhance plant salt tolerance by mechanisms such as maintenance of ion homeostasis, water and nutrient absorption, and selective uptake of salt ions [29]. Therefore, *Kamienskia* may also play a key role in the response of poplar forest ecosystems to drought and saline conditions.

Currently, approximately 900 million hectares of land is at risk of salinization worldwide as a result of natural and anthropogenic activities [52], as well as widespread saline–alkali soils all over China [53]. Previous research has demonstrated that the microbial activity, nutrient cycling, and health of soil can be negatively affected by soil salinity [54,55]. Moreover, soil salinity is reported to significantly alter the composition of microbial community diversity [55]. Our results likewise revealed that soil salinity regulated the species composition of AM fungi and their specialist and generalist subcommunities, mainly because plants, especially saline plants [31], can recruit specific soil microorganisms under salt stress [32]. For example, root-associated microorganisms can help plants to survive under saline stress [4], probably because soil salinity limits the uptake of water by microorganisms and plants [56]. In addition, salinity may cause high intracellular ion concentrations, which can upset the delicate osmotic balance and result in some toxicity, thereby exerting profound impacts on microbial growth [57], biomass, and activity [58,59]. Soil pH also stands out as a key determinant of the composition and variety within soil microbial ecosystems [60], which is inconsistent with our findings. It may be because *P. euphratica* is a pioneer tree species with good saline–alkali tolerance and suitable for highly saline–alkali environments, and soil pH has less effect than soil salinity when soil microorganisms are threatened by severe salt stress. Thus, this study highlights the important impact of soil salinity on AM fungal community composition and structure under saline–alkali stress.

### 4.2. Dynamic Balance of Stochastic and Deterministic Processes Shapes the Assembly of AM Fungal Community

It is essential to clarify the environmental factors that influence the balance between deterministic and stochastic processes that modify the assembly of microbial communities [61], and the assembly of an AM fungal community has been identified as key to understanding ecosystem functions [5,62]. Previous studies have concentrated on how the AM fungal community structure strikes a balance between deterministic and stochastic processes in forest [8], alpine meadow [63], and farmland [20]. The harsh ecological conditions in arid regions often limit species’ ability to be selected through environmental adaptation, thereby amplifying the effects of stochastic processes. It has been reported that biodiversity tends to be relatively low, and community establishment is more dependent on random dispersal or colonization than on environmental selection or interspecies competition in extremely arid environments [16,31]. A study has suggested that aerial dispersal is very common in AM fungi and is a major assembly process of AM fungal communities [64]. Moreover, AM fungi can spread actively by colonizing host plants and forming vast mycelial networks in the soil [21]. Additionally, Davison et al. [65] have demonstrated that AM fungi have a good dispersal capability, and environmental conditions are crucial for AM fungal community assembly.

In addition, this study found that the community assembly processes of generalists and specialists were equally dominated by stochastic processes, which is consistent with a study on Tibetan lake sediments [23] but contradicts our hypothesis. Previous studies have shown stronger deterministic processes in the assembly of specialist subcommunities and stronger stochastic processes in the assembly of generalist subcommunities [22]. However, the results of this study shed a different light on their relationships; that is, specialists were more susceptible to stochastic processes than generalists, particularly to dispersal limitation. This may be because the specialist subcommunity tends to have higher diversity than the generalist subcommunity. An earlier study has revealed that the community assembly of bacteria with high diversity is dominated by stochastic processes, while that of bacteria with low diversity is more strongly controlled by deterministic processes [66], because high diversity helps to enhance environmental tolerance and reduce sensitivity to biotic and abiotic selection pressures [67]. We also investigated the assembly processes of the AM fungal community at different soil salinity levels. Stronger deterministic processes were observed in the community assembly of AM fungi and their generalists and specialists under low and high soil salinity conditions, which is consistent with a study on the community assembly of *P. euphratica* rhizosphere bacteria [4]. This finding suggests that environmental filtering becomes more pronounced in extreme environments [68]. In other words, significant environmental stress tends to magnify the predictable processes that shape soil microbial communities [66]. Therefore, this study suggests that stochastic processes, such as dispersal limitation, stochastic colonization, and population fluctuations, dominate AM fungal community assembly under conditions of resource scarcity and environmental stress.

This study employed PLS-PM to elucidate how environmental variables, particularly soil salinity, impact the community assembly dynamics of AM fungi within a *P. euphratica* forest. First, altitude causes comprehensive changes in light, temperature, water, and soil factors, which is of great importance to biodiversity conservation, distribution patterns, and driving factors. Moreover, this study revealed the considerable influence of climatic variables, like AI, MAP, and PS, on AM fungal community assembly, particularly on generalist subcommunity assembly. This may be attributed to the fact that precipitation is a key driver of ecosystem processes in arid areas [69], and AI, MAP, and PS are closely related to the water status of *P. euphratica* forests. Moreover, as a key indicator of vegetation health and biomass [70], which partially reflects primary productivity [71], the NDVI is a key ecosystem attribute in the study of arid and semi-arid ecosystems and plays a vital role in understanding changes in ecosystem processes [72]. For example, there is a mutual dependence between soil water contents and NDVI, especially in arid and semi-arid areas [73]. In addition, results based on fertilization experiments have shown that soil microbial community succession is mainly driven by soil carbon and nitrogen contents, rather than by phosphorus contents [74]. This is because carbon and nitrogen are the basis for microbial growth, which is consistent with our results, further emphasizing the important role of carbon and nitrogen in AM fungal community assembly. Furthermore, soil bulk density influences porosity, structure, and water-retaining capacity, which further alters the supply of oxygen and nutrients for microorganisms to perform related ecological functions [75,76], affects microbial survival and activity, and, ultimately, modifies community assembly processes. These findings emphasize the role of soil salinity as a key environmental driver shaping the composition and assembly processes of AM fungal communities in desert riparian forests of extremely arid regions.

### 4.3. Stability of AM Fungal Network Under the Influence of Soil Salinity

The analysis of the ecological network showed that the stability of the AM fungal network exhibited a U-shaped relationship with increasing soil salinity, while the network complexity exhibited a hump-shaped relationship, which challenges the results of study on *P. euphratica* rhizosphere bacterial network [4]. Past studies have shown that competition for resources in root space can lead to fierce competition or even exclusion among microorganisms, but the ability of AM fungi to colonize does not affect coexistence [5]. However, some studies have suggested that the relationship between microbial interaction and stress gradient is hump-shaped [77], which is consistent with our results. This may be due to the fact that the positive indirect interaction does not increase synchronously or even decays under enhanced stress, which leads to the collapse of positive interaction [78,79]. It means that appropriate salt stress is beneficial to the complexity of AM fungal network. However, the network stability showed an opposite trend, and the mechanism may involve the following aspects: when soil salinity reaches the medium level, the accumulation of Na^+^ and Cl^−^ in the soil increases, resulting in an imbalance in plant ion homeostasis. Although AM fungi can maintain the ion ratio by regulating transporter genes, the microbial metabolic activity may be temporarily limited by salt toxicity, resulting in a decrease in network stability, while AM fungi may activate more complex stress mechanisms, such as enhancing antioxidant enzyme activity, accumulating osmotic adjustment substances, and regulating plant hormone signals, to adapt themselves to the environment under high soil salinity conditions [80], thereby restoring the stability of the AM fungal network.

## 5. Conclusions

This study focused on AM fungal communities in *P. euphratica* forests in extremely arid regions of northwest China and explored the community composition, assembly, and network of soil AM fungi, as well as their drivers. Our findings demonstrated that stochastic processes rather than deterministic processes dominated the community assembly of AM fungi. In addition, habitat generalists were more strongly influenced by deterministic processes than specialists, which makes it difficult to predict the changes in specialist subcommunities and their activities in the *P. euphratica* forest ecosystem. Moreover, stronger deterministic processes (variable selection) were observed under low and high soil salinity conditions. In addition, the network analysis showed that the fungal network complexity had a hump-shaped relationship with increasing soil salinity, while the network stability had a U-shaped relationship. Notably, soil salinity directly regulated the community assembly of AM fungi. This study highlights the importance of soil salinity as a key regulator of AM fungal community composition and assembly processes in arid ecosystems and provides a foundation for understanding the balance between stochastic and deterministic processes in extreme environments, furthering our knowledge of microbial ecology and resilience in the face of environmental challenges. However, this study focuses on the stable situation after the impact of the environment, and there are some limitations. Future research can further explore the response process of AM fungal community construction and its network characteristics to salt stress through long-term salt stress experiments or including more than one arid salty environment along natural salinity gradients to track dynamic changes in AM fungal community assembly processes and network characteristics over time.

## Data Availability

The AM fungi raw sequences used in this paper are available in the NCBI Sequence Read Archive under BioProject PRJNA1291137. Data can be downloaded in National Center for Biotechnology Information (NCBI) publicly accessible database.

## Supplementary Materials

The following supporting information can be downloaded at: https://www.mdpi.com/article/10.3390/microorganisms13081742/s1, Figure S1: Phylogenetic Mantel correlogram evaluating phylogenetic signal in the soil arbuscular mycorrhizal fungal communities sampled in this study; Figure S2: Genus composition of AM fungal communities in *Populus euphratica* forests; Figure S3: Nonmetric multidimensional scaling (NMDS) plot of soil arbuscular mycorrhizal fungal communities at different salinity levels based on the Bray–Curtis distance; Figure S4: Relationships between soil arbuscular mycorrhizal fungal community alpha diversity and soil salinity; Figure S5: Relationships between soil arbuscular mycorrhizal fungal community assembly processes (βNTI) and differences in soil salinity; Figure S6: A priori partial least squares path modeling (PLS-PM) of arbuscular mycorrhizal fungal community assembly processes; Figure S7: Relationships between soil arbuscular mycorrhizal fungal community alpha diversity and positive cohesion; Figure S8: Relationships between soil arbuscular mycorrhizal fungal community alpha diversity and negative cohesion; Figure S9: AM fungal networks at different salinity levels; Table S1: Principal component analysis of arbuscular mycorrhizal fungal network stability indices; Table S2: Degree of influence of environmental variables on soil arbuscular mycorrhizal fungal (total) community assembly processes; Table S3: Degree of influence of environmental variables on soil arbuscular mycorrhizal fungal (specialist) community assembly processes; Table S4: Degree of influence of environmental variables on soil arbuscular mycorrhizal fungal (generalist) community assembly processes.
