# Supplementary material for: Soil Salinity Drives the Arbuscular Mycorrhizal Fungal Generalists and Specialists Subcommunity Assembly in Extremely Dryland Forest in China
Source: Microorganisms. 2025 Jul 25;13(8):1742. doi: 10.3390/microorganisms13081742 (PMC12388618; doi:10.3390/microorganisms13081742)
Supplement: Supplementary file 1 [file microorganisms-13-01742-s001.zip › microorganisms-3751107-supplementary.pdf]

Supplementary Materials

Tables and Figures

Table S1 Principal component analysis of arbuscular mycorrhizal fungal network stability indices.

Table S2 Degree of influence of environmental variables on soil arbuscular mycorrhizal fungal (total) community assembly processes.

Table S3 Degree of influence of environmental variables on soil arbuscular mycorrhizal fungal (specialist) community assembly processes.

Table S4 Degree of influence of environmental variables on soil arbuscular mycorrhizal fungal (generalist) community assembly processes.

Figure S1 Phylogenetic Mantel correlogram evaluating phylogenetic signal in the soil arbuscular mycorrhizal fungal communities sampled in this study.

Figure S2 Genus composition of AM fungal communities in *Populus euphratica* forests.

Figure S3 Nonmetric multidimensional scaling (NMDS) plot of soil arbuscular mycorrhizal fungal communities at different salinity levels based on the Bray-Curtis distance.

Figure S4 Relationships between soil arbuscular mycorrhizal fungal community alpha diversity and soil salinity.

Figure S5 Relationships between soil arbuscular mycorrhizal fungal community assembly processes (βNTI) and differences in soil salinity.

Figure S6 A priori partial least squares path modeling (PLS-PM) of arbuscular mycorrhizal fungal community assembly processes.

Figure S7 Relationships between soil arbuscular mycorrhizal fungal community alpha diversity and positive cohesion.

Figure S8 Relationships between soil arbuscular mycorrhizal fungal community alpha diversity and negative cohesion.

Figure S9 AM fungal networks at different salinity levels.

Table S1 Principal component analysis of arbuscular mycorrhizal fungal network stability indices.

Salinity(mS/cm)	**PC1**	PC2
	**63.10%**	36.90%
0-1	**0.348**	0.702
1-2	**-1.574**	0.968
2-4	**-0.945**	-1.494
4-7	**0.604**	-0.149
7-11	**1.567**	-0.027

Note: The network stability was evaluated by robustness and vulnerability through principal component analysis. And subsequent data analysis used the PC1 axis.

Table S2 Degree of influence of environmental variables on soil arbuscular mycorrhizal fungal (total) community assembly processes.

Category	Variable	Direct effect	Indirect effect	Total effect
Altitude	Altitude	-0.076	0.005	-0.071
Climate	AI, MAP, PS	0.000	0.000	0.000
Vegetation	NDVI	-0.011	0.000	-0.011
Soil bulk density	BD	0.016	0.025	0.041
Salinity	Salinity	0.115	0.002	0.117
Soil nutrient content	SOC, TN	0.014	-	0.014

Note: AI, aridity index; MAP, mean annual precipitation; PS, precipitation seasonality; NDVI, normalized difference vegetation index; BD, Soil bulk density; SOC, soil organic carbon content; TN, total nitrogen content.

Table S3 Degree of influence of environmental variables on soil arbuscular mycorrhizal fungal (specialist) community assembly processes.

Category	Variable	Direct effect	Indirect effect	Total effect
Altitude	Altitude	-0.103	-0.031	-0.134
Climate	AI, MAP, PS	-0.059	0.006	-0.053
Vegetation	NDVI	-0.031	0.003	-0.028
Soil bulk density	BD	-0.003	0.021	0.018
Salinity	Salinity	0.068	0.006	0.075
Soil nutrient content	SOC, TN	0.037	-	0.037

Note: AI, aridity index; MAP, mean annual precipitation; PS, precipitation seasonality; NDVI, normalized difference vegetation index; BD, Soil bulk density; SOC, soil organic carbon content; TN, total nitrogen content.

Table S4 Degree of influence of environmental variables on soil arbuscular mycorrhizal fungal (generalist) community assembly processes.

Category	Variable	Direct effect	Indirect effect	Total effect
Altitude	Altitude	-0.043	0.187	0.144
Climate	AI, MAP, PS	0.208	0.071	0.279
Vegetation	NDVI	-0.087	0.028	-0.060
Soil bulk density	BD	0.034	0.075	0.109
Salinity	Salinity	0.133	0.020	0.153
Soil nutrient content	SOC, TN	0.192	-	0.192

Note: AI, aridity index; MAP, mean annual precipitation; PS, precipitation seasonality; NDVI, normalized difference vegetation index; BD, Soil bulk density; SOC, soil organic carbon content; TN, total nitrogen content.

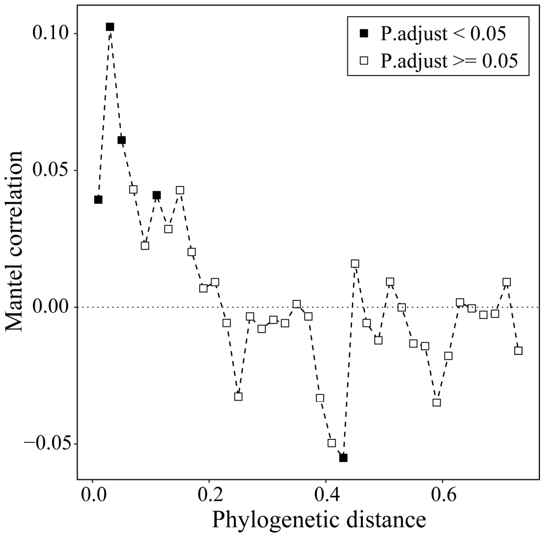

Figure S1 Phylogenetic Mantel correlogram evaluating phylogenetic signal in the soil arbuscular mycorrhizal fungal communities sampled in this study. The plot relates Pearson correlation coefficients to phylogenetic distances. symbols represent 37 bins divided by a phylogenetic distance of 0.02, with solid symbols indicating significant phylogenetic signals in OTU ecological niches and hollow symbols for non-significant associations. The analysis reveals significant phylogenetic signal.

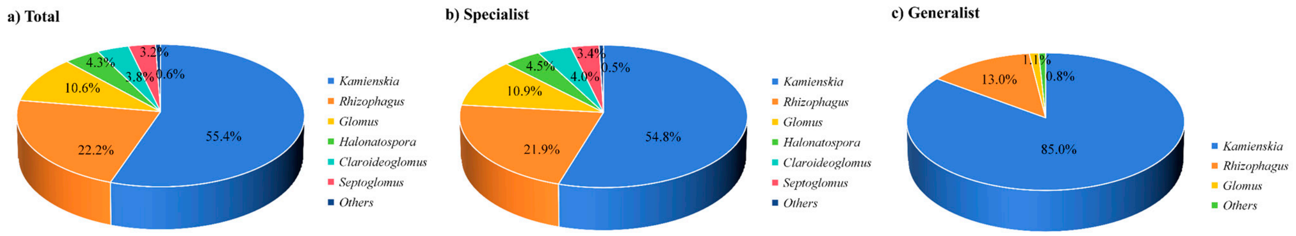

Figure S2 Genus composition of AM fungal communities in *Populus euphratica* forests. a) Total of arbuscular mycorrhizal fungal; **b)** Specialist of arbuscular mycorrhizal fungal; **c)** Generalist of arbuscular mycorrhizal fungal. Genus with an average relative abundance of less than 1% is classified as “Others”.

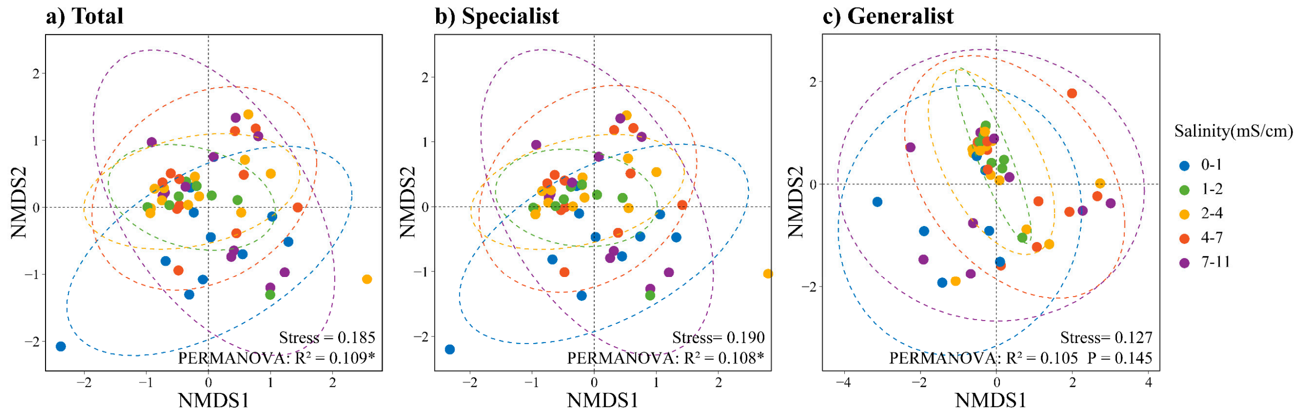

Figure S3 Nonmetric multidimensional scaling (NMDS) plot of soil arbuscular mycorrhizal fungal communities at different salinity levels based on the Bray-Curtis distance. a) Total of arbuscular mycorrhizal fungal; **b)** Specialist of arbuscular mycorrhizal fungal; **c)** Generalist of arbuscular mycorrhizal fungal. *, $P < 0.05$.

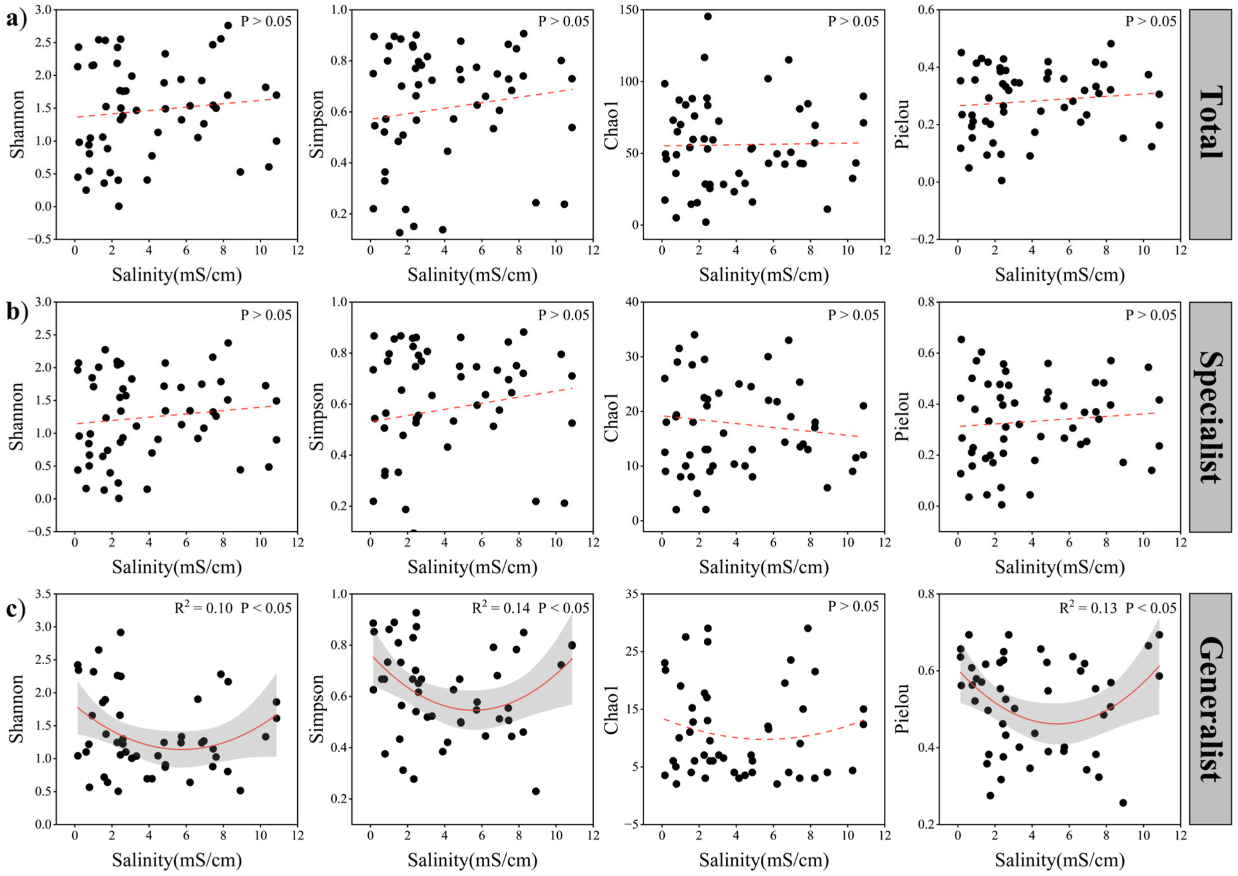

Figure S4 Relationships between soil arbuscular mycorrhizal fungal community alpha diversity and soil salinity. a) Total of arbuscular mycorrhizal fungal; **b)** Specialist of arbuscular mycorrhizal fungal; **c)** Generalist of arbuscular mycorrhizal fungal. Four indices, including Shannon, Simpson, Chao1, and Pielou, are selected to characterize alpha diversity. The results of linear regression analysis were presented. Correlations with $P > 0.05$ are expressed as a dotted line. Shaded bands are 95% CIs.

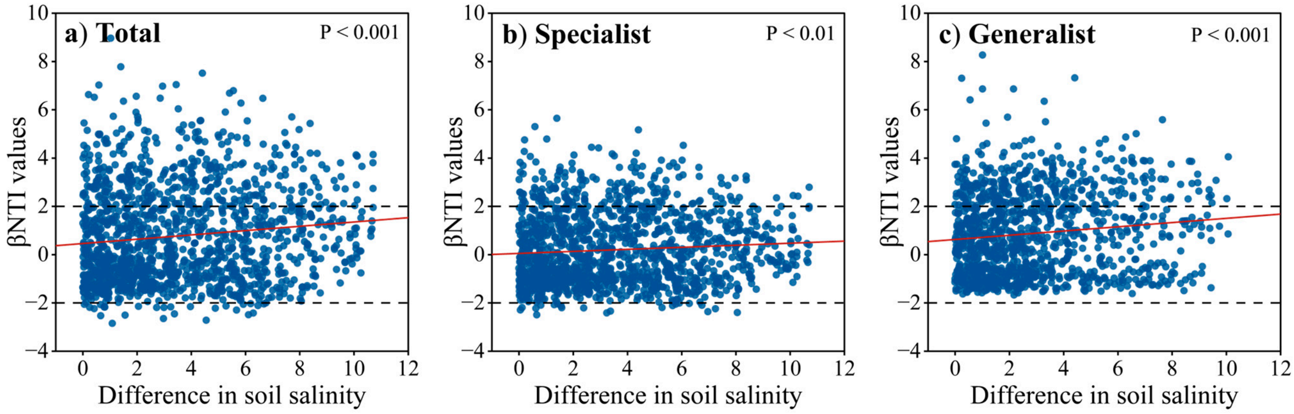

Figure S5 Relationships between soil arbuscular mycorrhizal fungal community assembly processes (βNTI) and differences in soil salinity. a) Total of arbuscular mycorrhizal fungal; **b)** Specialist of arbuscular mycorrhizal fungal; **c)** Generalist of arbuscular mycorrhizal fungal. Linear regression models (shown as red lines) and associated correlation coefficients are provided on each panel. Horizontal dashed lines indicate the βNTI significance thresholds of +2 and −2.

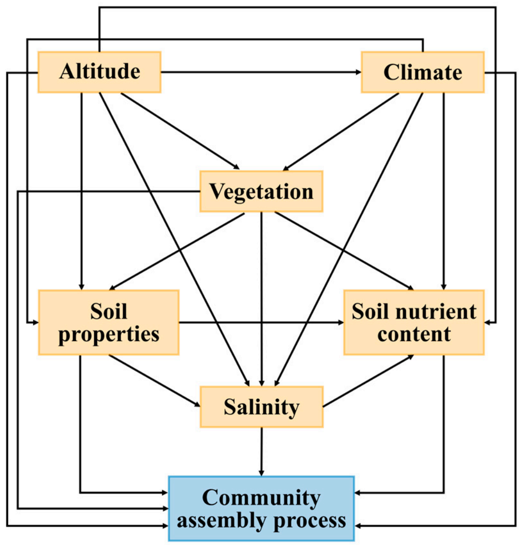

Figure S6 A priori partial least squares path modeling (PLS-PM) of arbuscular mycorrhizal fungal community assembly processes. Climate, AI (aridity index), MAP (mean annual precipitation), MAT (mean annual temperature), and PS (precipitation seasonality); vegetation, NDVI (normalized difference vegetation index) and PR (plant richness); soil properties, BD (bulk density) and SMC (soil moisture content); soil nutrient content, SOC (soil organic carbon content), TP (total phosphorus content), and TN (total nitrogen content). Variables with loading less than 0.7 were excluded, and the remaining environmental variables were shown in Figure 5 of revised manuscript.

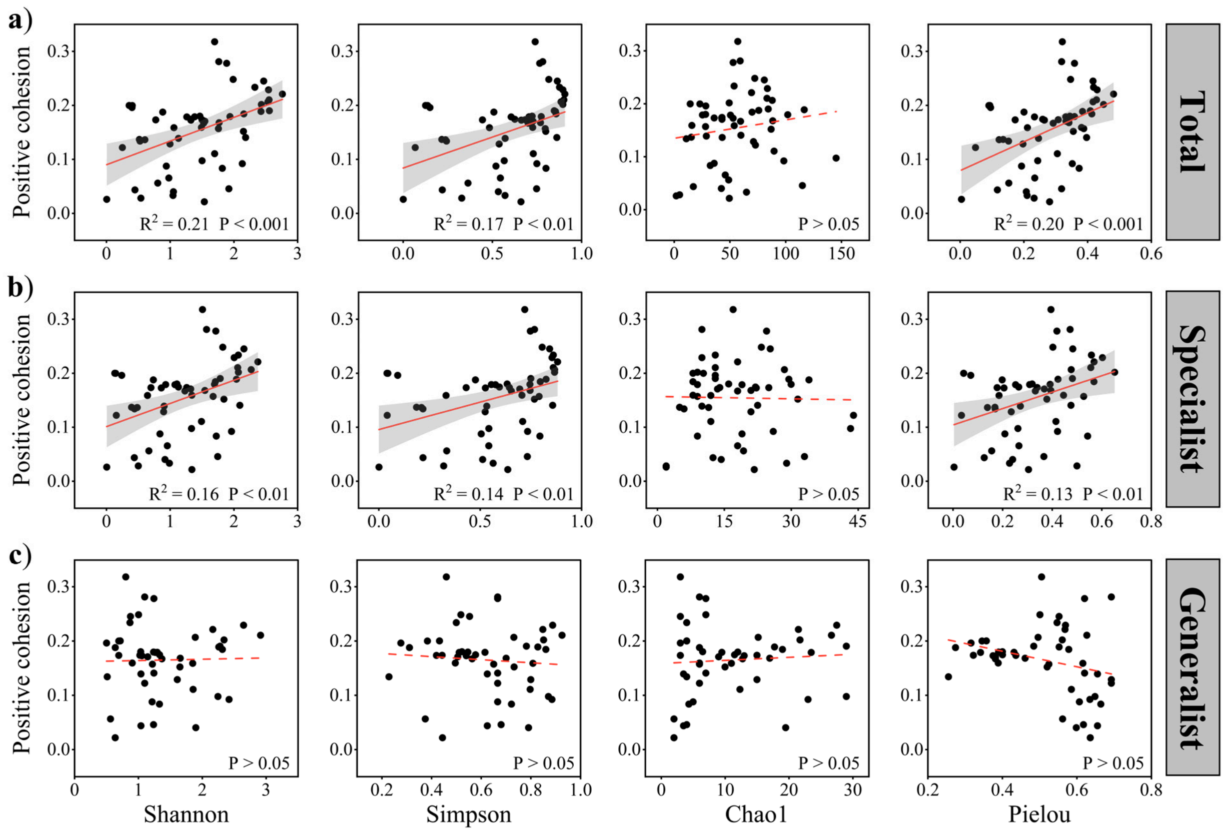

Figure S7 Relationships between soil arbuscular mycorrhizal fungal community alpha diversity and positive cohesion. a) Total of arbuscular mycorrhizal fungal; **b)** Specialist of arbuscular mycorrhizal fungal; **c)** Generalist of arbuscular mycorrhizal fungal. Four indices, including Shannon, Simpson, Chao1, and Pielou, are selected to characterize alpha diversity. The results of linear regression analysis were presented. Correlations with $P > 0.05$ are expressed as a dotted line. Shaded bands are 95% CIs.

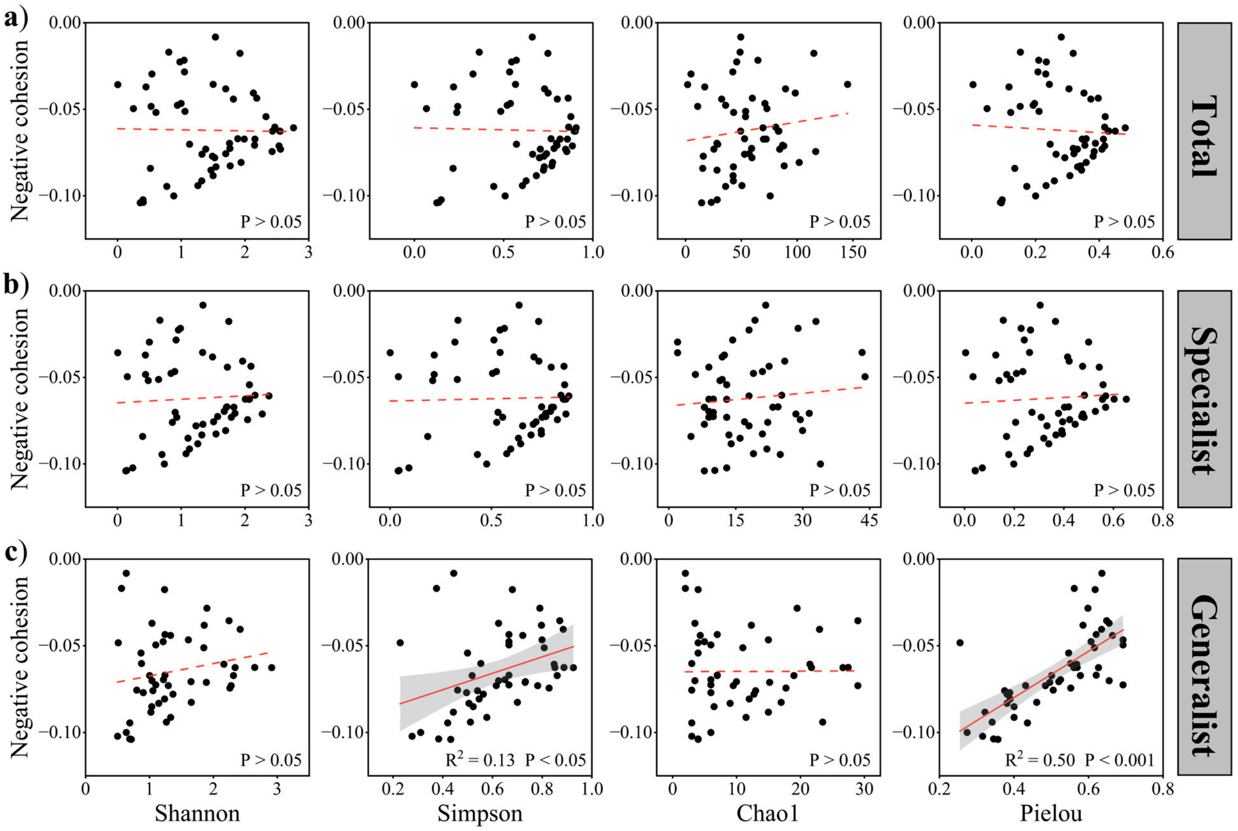

Figure S8 Relationships between soil arbuscular mycorrhizal fungal community alpha diversity and negative cohesion. a) Total of arbuscular mycorrhizal fungal; **b)** Specialist of arbuscular mycorrhizal fungal; **c)** Generalist of arbuscular mycorrhizal fungal. Four indices, including Shannon, Simpson, Chao1, and Pielou, are selected to characterize alpha diversity. The results of linear regression analysis were presented. Correlations with P > 0.05 are expressed as a dotted line. Shaded bands are 95% CIs.

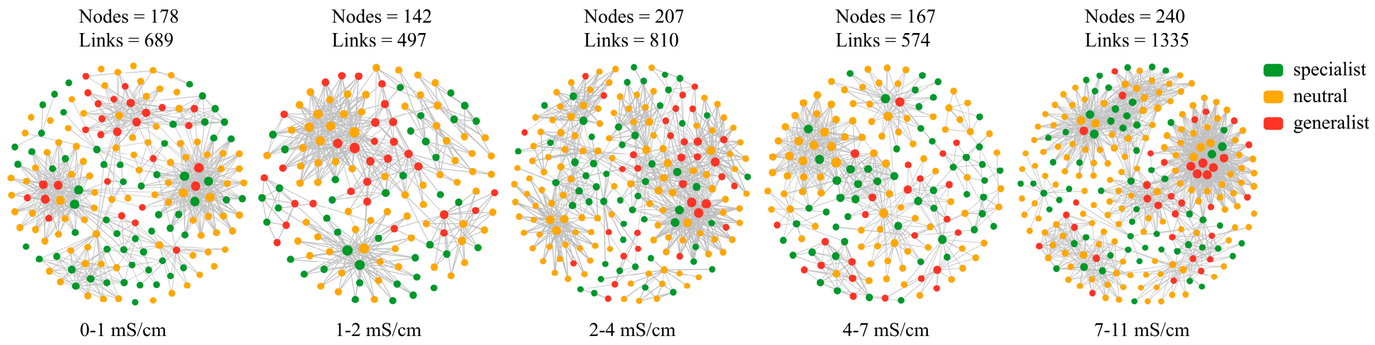

Figure S9 AM fungal networks at different salinity levels. AM fungal network with nodes colored based on OTUs. Specialist, generalist and neutral AM fungi.
